# Prevalence of PTSD and Depression among Junior Middle School Students in a Rural Town Far from the Epicenter of the Wenchuan Earthquake in China

**DOI:** 10.1371/journal.pone.0041665

**Published:** 2012-07-23

**Authors:** Wei Wang, Wei Fu, Jin Wu, Xian-cang Ma, Xue-li Sun, Yi Huang, Kenji Hashimoto, Cheng-ge Gao

**Affiliations:** 1 Department of Psychiatry, First Affiliated Hospital of Medical College of Xi'an Jiaotong University, Xi'an, China; 2 Mental Health Center, West China Hospital, Sichuan University, Chengdu, China; 3 Division of Clinical Neuroscience, Chiba University Center for Forensic Mental Health, Chiba, Japan; Rikagaku Kenkyūsho Brain Science Institute, Japan

## Abstract

**Context:**

On May12^th^ 2008, a devastating earthquake measuring 8.0 on the Richter scale, struck Wenchuan county and surrounding areas in China. The prevalence of mental illness among children and adolescents in a rural town far from the earthquake epicenter is unknown.

**Objective:**

To assess the prevalence of posttraumatic stress disorder (PTSD) and depression among junior middle school students in a rural town Ningqiang county, 327 km from the earthquake epicenter.

**Design, Setting, and Participants:**

A population-based mental health survey was conducted in March, 2009.

**Main Outcome Measure:**

Survey Self-designed General Condition Survey Scale, Children's Revised Impact of Event Scale (CRIES-13), and the Depression Self-rating Scale for Children (DSRSC) were used to sample 1,841 junior middle school students in Ningqiang county, ten months after the Wenchuan earthquake.

**Results:**

The prevalence rate of a high-risk for PTSD was 28.4%, with 32.7% among females, 23.8% among males (female vs. male, p<0.001), 38.6% in the severe exposure group and 24.3% in the mild exposure group (severe vs. mild exposure, p<0.001). For depressive symptoms, the overall prevalence was 19.5%, with 24.0% among females, 14.7% among males, 24.5% in the severe exposure group and 17.5% in the mild exposure group (female vs. male, p<0.001; severe vs. mild exposure, p<0.001, respectively). In multivariate analysis, factors such as “having felt despair”, or “danger” and “having own house destroyed or damaged” were significantly associated with PTSD symptoms. Female gender and delayed evacuation in females, and earthquake related experiences in males were significantly associated with depression.

**Conclusion:**

Traumatic events experienced during the earthquake were significantly associated with symptoms of PTSD and depression in children and adolescents, ten months after the Wenchuan earthquake. These data highlight a need for mental health services for children and adolescents in rural areas, far from earthquake epicenters.

## Introduction

It is well established that natural disasters, such as earthquakes lead to increased prevalence of mental illness in the range of 5 to 40%, although this prevalence is dependent upon the disaster severity and population exposure [1]–[4]. Post-traumatic stress disorder (PTSD) is the most frequent and debilitating psychological disorder that occurs after traumatic events and natural disasters.

**Figure 1:**
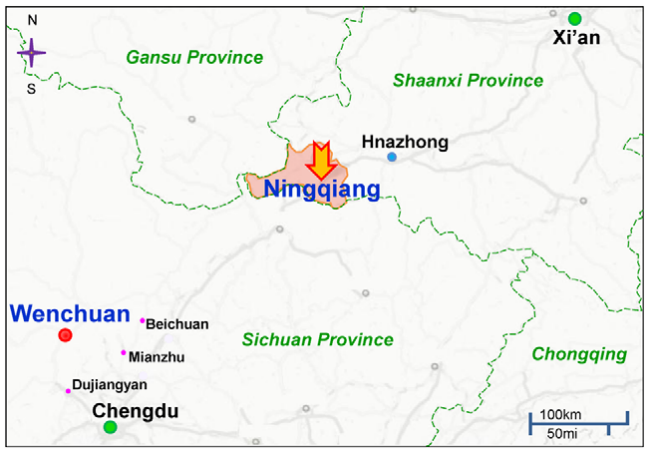
Geographical location of the Ningqiang study cohort relative to the 2008 Wenchuan Earthquake in China. Chengdu and Xi'an are the capital of the Sichuan province and Shaanxi province, respectively. The epicenter Wenchuan is 80 kilometers west-northwest of Chengdu. Ningqiang located in the southwest of the Shaanxi province is the nearest town in the Shaanxi province to the epicenter (Wenchuan).

On May 12, 2008, a devastating earthquake measuring 8.0 on the Richter scale, occurred in the Wenchuan county of Sichuan Province, in southwest China (**Figure 1**). This earthquake was the most destructive natural disaster in modern Chinese history. More than 90,000 people were reported dead or missing, and more than 400,000 people were injured [5]. This disaster not only caused huge damage to people's personal and economic wellbeing, but also left indelible psychological trauma in survivors across the Sichuan, Shaanxi and Gansu Provinces [6]–[9]. Some reports show that children and adolescents are more susceptible to mental health problems after catastrophic events, such as earthquakes, although debate still exists over these findings [10]–[13]. It is, therefore, important to study the mental health of children and adolescents in disaster areas, in order to provide appropriate mental health support for survivors of natural disasters.

Many studies conducted after the Wenchuan earthquake focused on survivors close to the epicenter and its surrounding areas [5], [8], [14]. In contrast, there are no studies reporting on the mental health problems of survivors in rural areas, far from the epicenter, such as Wenchuan county. In this study, we examined the mental health problems of children and adolescents in a rural area (Ningqiang county, Shaanxi Province, China) located approximately 327 km from the epicenter, 10 months after the earthquake (**Figure 1**). Using the psychiatric assessment scale for children, the mental health status of a large sample (n = 1,841) of junior middle school students from Ningqiang county was evaluated. The complete and accurate survey data, encompassing a post-disaster high risk population with mental health problems, was screened and analyzed to provide future psychological assistance and interventions supported by data, for affected populations.

## Methods

### Study design

Ningqiang county (Ningqiang), located in the southwest of Shaanxi Province of China, is a small rural area under the jurisdiction of Hanzhong city, and lies approximately 327 km from the Wenchuan earthquake epicenter (**Figure 1**). This county was selected for study because it was the most severely affected area in Shaanxi Province during the earthquake. The county suffered over 500 collapsed buildings, seven deaths and several hundred injured citizens. The county is a national-level poverty-stricken region in China.

The initial random data included 2,048 students from Yifu Junior Middle School (grades 7–9), the largest junior middle school in Ningqiang county. This school encompasses almost all junior middle school-aged children within the county and around the town, excluding several remote mountain areas. Ordinary cluster sampling methodology was used, and the survey was performed in school, during classroom time. Of all surveyed subjects, 1,967 (96.0%) completed the investigation. When invalid questionnaires were removed (based on pre-established exclusion criteria), the final sample, consisted of 1,841 (89.9%) valid questionnaires. This final sample comprised 896 males (48.7%) and 945 females (51.3%), with a mean age of 14.2±1.2 years and range from 11 to 20 years. This large age range was due to two main phenomena. Firstly, children from low-income families, particularly those from mountainous areas often enroll in school at a later age. Secondly, pupils who fail year-end examinations are required to repeat the grade, even in elementary and junior middle school, leading to older than average pupils. Of the subjects, 1827 (99.2%) were Han Chinese and 14 (0.8%) were ethnic minorities (**Table 1**). All surveyed subjects spoke Chinese.

**Table 1:** Demographic characteristics and earthquake experiences among junior middle school students in Ningqiang county.

	Number of students (%)
**Gender**	
Male	896 (48.7)
Female	945 (51.3)
**Ethnicity**	
Han	1827 (99.2)
Ethnic minorities	14 (0.8)
**Age (mean age of 14.2±1.2)**	
<12	113 (6.1)
13	471 (25.6)
14	553 (30.0)
15	456 (24.8)
16	222 (12.1)
>17	26 (1.4)
**Grade**	
7	604 (32.8)
8	657 (35.7)
9	580 (31.5)
**Personal experiences during the earthquake**	
Were buried or trapped	11 (0.6)
Injured, hospitalized or required surgery	271 (1.5)
Felt danger	1053 (57.2)
Felt despair	140 (7.8)
Family members killed or injured	45 (2.4)
Own house destroyed or severely damaged	140 (7.8)
Witnessed injury/death	204 (11.1)
Witnessed building collapse	172 (9.3)
**Timing to move into tents**	
** <2 days**	1322 (71.8)
** 2–3 days**	151 (8.2)
** >3 days**	165 (9.0)
**Current living area**	
Original residence	1731 (94.0)
Moved to a new place	110 (6.0)
**Living environment (at the time of the survey)**	
Own home	1600 (89.0)
Other person's home	130 (7.2)
Tent	59 (3.3)
Board room	8 (0.5)
**Timing of resumption of classes**	
<1 week	116 (6.0)
1–2 weeks	55 (3.0)
3–4 weeks	434 (23.6)
>4 weeks	1241 (67.4)

This study was conducted with the permission of the National Science and Technology Ministry, and the survey protocol was approved by the Human Research Ethics Committee of Xi'an Jiaotong University (Permit Number: 2008LLKZ128). Permission was also obtained from the Education Bureau of Ningqiang county and School Boards. The interview procedures were explained to students, and parents and teachers gave written consent for children to participate. Written informed consents were obtained from all surveyed subjects. The investigation was conducted by 8 interviewers, including two psychiatrists with at least 5 years of experience in general psychiatric practice and six psychological consultants. All interviewers participated in a 2-day training program to ensure appropriate understanding and consistent application of the questionnaires and scales.

### Data collection

The data were collected in March 2009, ten months after the earthquake. The investigation process was completed within one day. The initial assessments were made using self-report measures, including the General Condition Information, Children's Revised Impact of Event Scale (CRIES), and Depression Self-Rating Scale for Children (DSRSC).

#### General condition information

Demographic data included age, gender and grade. Personal experience during the earthquake was assessed using the following questions:

- Whether you were trapped;
- Whether you were injured, hospitalized or required surgery;
- Whether you felt danger or despair;
- Whether family members were killed or injured;
- Whether own house was destroyed or severely damaged;
- Whether you witnessed a death or injury to someone (family members, friends, or strangers), or witnessed a collapse of buildings.

According to the degree of exposure during the earthquake, surveyed subjects were divided into two groups:

1. Severe exposure group: students in this group experienced at least one of the following five exposures: 1) trapped in a collapsed buildings; 2) suffered injury, hospitalization or surgery; 3) family members killed or injured; 4) own house destroyed or severely damaged and uninhabitable; 5) witnessed a death or injury to someone (family member, friend, or stranger) or collapsed buildings.
2. Mild exposure group: did not experience any of the above five exposures.

#### Children's Revised Impact of Event Scale (CRIES-13)

CRIES-13 is a newly developed 13-item scale adapted from the impact of event scale [15]. It is suitable for screening populations with high risk of PTSD from large samples of children who have experienced different traumatic events [16]. The total scores of the response on this scale indicate the severity of posttraumatic stress reactions, with a range from 0 to 65. CRIES-13 includes three subscales: Intrusion (four items), Avoidance (four items) and Arousal Symptoms (five new items). The Chinese version of CRIES-13 translated by Ma and So [17], was used in this study. The reliability and validity of the Chinese version of CRIES-13 was evaluated by Zhang et al. [18]. A cut-off score of 30 was recommended to define probable PTSD [19], and it has been used in clinical studies as the most efficient cut-off for discriminating cases of PTSD [18], [20], [21]. Here, using this method the surveyed population was divided into normal and high-risk PTSD groups.

#### Depression Self-Rating Scale for Children (DSRSC)

DSRSC [22] is widely used to measure children's depressive symptoms. This scale consists of 18 items, and possible scores range from 0 to 36. Su et al. [23] created a Chinese version of the DSRSC and have demonstrated satisfactory reliability and validity. A total score of 15 has been suggested as the cut-off point to identify depressive disorders in Chinese children, with acceptable sensitivity and specificity [23]. In this study, the surveyed students were divided into normal and depression high-risk groups using this method.

### Data analysis

Prevalence of PTSD and depression was calculated and analyzed using SPSS version 18.0 (SPSS Inc, Chicago, IL). The total scores and various subscale scores from CRIES-13 and DSRSC were computed according to each respective manual, and frequencies, percentages, mean value and standard deviations or standard errors were calculated. Chi-square tests were used to evaluate differences in categorical data, and Student *t*-tests were used to evaluate differences in continuous variables, when the data was of normal distribution and homogeneity of variance. If the normal distribution and homogeneity of variance were not met, Wilcoxon rank sum test was used instead. Pearson correlation was used to analyze the interrelationship between the CRIES-13 and DSRSC scales.

Logistic regression models were used to identify factors influencing CRIES and DSRSC scores. The score groupings of CRIES and DSRSC were taken as the dependent variables while gender and age characteristics, related experiences during the earthquake and the situation after the earthquake, were selected as independent variables. Multivariate models were set up using the Forward Selection (Likelihood Ratio) method. In addition, Chi-square automatic interaction detector (CHAID) decision trees were established to screen the risk indicators in high risk subjects with PTSD and depression. The decision tree models were fitted for categorical variable PTSD and depression. The total scores of CRIES and DSRSC were taken as the result variable, and independent variables were the same as mentioned previously. The optimal minimum parent and child nodes were set up to 100 and 50, respectively, and a maximum growth depth of 4 layers was selected in the final tree structure (α splitting  =  α, and merging  = 0.05.)

All independent variables with an α value of 0.05 or lower in bivariate analysis were entered into models, and the removal standard was α ≥0.10. For all analyses, *p* values of less than 0.05 were considered statistically significant.

## Results

On site, 2,048 questionnaires were issued, 1,967 were collected, and of these, 1,841 were evaluable. This gave a response rate of 96.0% and an effective response rate of 89.9%. Of the surveyed subjects, 7.8% had suffered destruction or damage to their house; 2.4% had family members killed or injured; 1.5% suffered injury, or were hospitalized or had surgery and 0.6% were buried or trapped in a collapsed building. The demographic characteristics, including the living environment after earthquake, are shown in **Table 1**.

Surveyed subjects were divided into two groups according to their experiences during the earthquake, for statistical analysis purposes. The severe exposure group totaled 522 students (28.4%) including 241 males (46.1%) and 282 females (53.9%), with a mean age of 14.17±1.14. The mild exposure group totaled 1319 students (71.6%) and included 655 males (49.7%) and 663 females (50.3%), with a mean age of 14.15±1.19. There were no differences between the mild and severe exposure groups (gender: χ^2^ = 1.9599, p = 0.162; age: χ^2^ = 2.8317, p = 0.726).

### Estimated high risk of PTSD

Normality testing indicated that the total CRIES-13 score did not meet a normal distribution pattern in this study (W = 0.984, p<0.001). The mean scores on CRIES-13 including total score and three subscales scores (intrusion, avoidance and arousal) were 22.74±11.85, 7.14±4.78, 7.32±5.16 and 8.28±5.23, respectively (**Table 2**). All scores were significantly higher in females and in the severe exposure group, than in males (23.99 vs. 21.41, p<0.001; 7.64 vs. 6.60, p<0.001; 7.62 vs. 7.00, p<0.05 and 8.73 vs. 7.81, p<0.001, respectively) and in the mild exposure group (25.76 vs. 21.53, p<0.001; 8.16 vs. 6.73, p<0.001; 8.15 vs. 6.99, p<0.001; 9.46 vs. 7.82, p<0.001, respectively) (**Table 2**).

**Table 2:** Total and subscales scores of CRIES-13 in different gender and exposure degrees.

	Total score (range)	Intrusion (range)	Avoidance (range)	Arousal (range)
**Overall (n = 1841)**	22.74±11.85 (0 ∼ 63)	7.14±4.78 (0 ∼ 20)	7.32±5.16 (0 ∼ 20)	8.28±5.23 (0 ∼ 25)
**Gender**				
Male(n = 896)	21.41±11.68	6.60±4.65	7.00±5.16	7.81±5.07
Female (n = 945)	23.99±11.89	7.64±4.84	7.62±5.14	8.73±5.34
Z	−4.697***	−4.747***	−2.625*	−3.757***
**Exposure degree**				
Mild (n = 1319)	21.53±11.69	6.73±4.71	6.99±5.15	7.82±5.14
Severe (n = 522)	25.76±11.72	8.16±4.80	8.15±5.09	9.46±5.27
Z	−7.193***	−6.090***	−4.485***	−6.288***

Data represent the mean ± S.D.

* *p*<0.05, ***p<0.001.

Using a cut-off score of 30 on CRIES-13 (total score), 522 (28.4%) students were estimated high risk for PTSD (total score ≥30). As presented in **Table 3**, the estimated prevalence of a high-risk for PTSD was significantly higher in females and the severe exposure group compared with males and the mild exposure group (32.7% vs. 23.8%, χ^2^ = 18.04, p = 0.001; and 38.6% vs. 24.3%, χ^2^ = 37.92, p<0.001, respectively). There was no difference in the distribution of a high-risk for PTSD with age (χ^2^ = 10.30, p = 0.067).

**Table 3:** Proportion of population at high risk for PTSD in different genders and exposure degrees*.

Variable	No. of Normal (%)	No. of high-risk PTSD (%)	*χ* ^2^	*p*
**Gender**			18.04	<0.001
Male (n = 896)	683 (76.23)	213 (23.77)		
Female (n = 945)	636 (67.30)	309 (32.70)		
**Exposure degree**			37.92	<0.001
Mild exposure group (n = 1319)	998 (75.72)	320 (24.28)		
Severe exposure group (n = 522)	321 (61.38)	202 (38.62)		

* Normal: Total CRIES-13 score <30; high risk PTSD: total CRIES-13 score ≥30.

Logistic Regression Analysis was performed to analyze multivariate factors affecting PTSD after the earthquake (**Table 4**). The highest estimated prevalence of a high-risk for PTSD was found in students who had felt despair (OR [95% CI]: 5.0 [2.6–9.9], p = 0.000). Furthermore, high prevalence of a high-risk for PTSD was also found in students whose house had been destroyed or severely damaged (OR [95% CI]: 2.9 [1.8–4.7]. These students were most likely to develop PTSD. A significant difference was also observed in gender (p = 0.000), with the risk of PTSD in females being 1.7 times that of males.

**Table 4:** Logistic regression analysis of PTSD-related factors.

Variable	β estimate value	OR	OR 95% CI		*p* value	
			Lower	Upper		
**Gender (n = 1841)^ a^**	0.501±0.131	1.651	1.276	2.135	0.000	
**Age (n = 1841)**	0.133±0.055	1.142	1.025	1.272	0.016	
**Whether felt danger (n = 1689)^b^**						
No, did feel danger (n = 633)		1.000				
Yes, but had hope (n = 1053)	0.788±0.147	2.198	1.647	2.934	0.000	
Yes, and felt despair (n = 140)	1.617±0.346	5.037	2.559	9.916	0.000	
**Witness earthquake related events (1673)^c^**						
No (n = 1297)		1.000				
Injury/death (n = 204)	0.793±0.191	2.211	1.520	3.216	0.000	
Building collapse (n = 172)	0.643±0.194	1.903	1.302	2.782	0.001	
**Own house damaged (n = 1801)^d^**						
No (n = 608)		1.000				
Yes, but inhabitable (n = 1053)	0.685±0.149	1.984	1.480	2.659	0.000	
Yes, uninhabitable/destroyed (n = 140)	1.071±0.248	2.917	1.795	4.740	0.000	

Data represent the mean ± S.E.

a) compared with males; b) compared with not feeling danger; c) compared with not witnessing earthquake related events; d) compared with no damage to own house. OR: Odds ratio.

### Estimated high risk of depression

Normality testing showed that total scores of DSRSC did not meet the normal distribution (W = 0.973, p<0.001). Total scores on DSRSC ranged from 0 (0.33%) to 30 (0.11%) with a mean of 10.36±5.16. On average, females scored significantly higher than males (11.12±5.34 vs. 9.54±4.83, *p*<0.001). Predictably, significantly higher DSRSC scores were found in the severe exposure group compared with the mild exposure group (11.26±5.10 vs. 10.00±5.14, *p*<0.001). There were no differences related to age (χ^2^ = 7.65, *p* = 0.176). Using 15 as a cut-off value, 19.5% (359) of students would be considered as having depressive symptoms (total score ≥15). As shown in **Table 5**, females comprise 24.02% on the proportion of the population with a high-risk for depression, and this prevalence was significantly higher than that in males (14.7%) (χ^2^ = 25.28, p<0.001). Additionally, the estimated prevalence of a high risk for depression in the severe exposure groups, was 24.47%, significantly higher than in the mild exposure groups (17.5%) (χ^2^ = 11.51, p = 0.001). There were no differences in the distribution of depression risk with age (χ^2^ = 3.60, p = 0.609).

**Table 5:** Proportion of population with high risk for depression in different genders and exposure degrees.*

Variable	No. of Normal (%)	No. of Depression high-risk (%)	*χ* ^2^	*p* value
**Gender**			25.28	<0.001
Male (n = 896)	764 (85.3)	132 (14.73)		
Female (n = 945)	718 (76.0)	227 (24.02)		
**Exposure degree**			11.51	<0.001
Mild exposure group (n = 1319)	1087 (82.5)	231 (17.53)		
Severe exposure group (n = 522)	395 (75.5)	128 (24.47)		

* Normal: Total DSRSC score <15; Depression high-risk: Total DSRSC score ≥ 15.

Results of Logistic Regression analysis indicated the highest prevalence of a high risk for depression in students who had felt despair. These students were most likely to develop depression (OR [95% CI]: 3.5 [1.7–7.0], p = 0.000) (**Table 6**). Significant differences were observed in gender (p = 0.000), with the risk of depression in females being 1.8 times that of males. In contrast, an inverse correlation was found between high depression risk students and the elapsed time before the resumption of classes. The OR value was lower in students who resumed classes no earlier than three to four weeks after their trauma (OR [95% CI]: 0.36 [0.19–0.68], p = 0.001, 0.30 [0.17–0.54], p = 0.000, respectively).

**Table 6:** Logistic regression analysis results of depression-related factors.

Variable	β estimate value	OR	OR 95% CI		*p* value
			Lower	Upper	
**Gender (1841) ^a^**	0.602±0.151	1.826	1.359	2.453	0.000
**Whether felt danger (n = 1689)^b^**					
No, did not feel danger (n = 633)		1.000			
Yes, but had hope (n = 1053)	0.247±0.159	1.280	0.937	1.748	0.121
Yes, and felt despair (n = 140)	1.258±0.353	3.518	1.760	7.032	0.000
**Witness to earthquake related events (n = 1673)^c^**					
No (n = 1297)		1.000			
Injury/death (n = 204)	0.317±0.228	1.373	0.878	2.147	0.165
Building collapse (n = 172)	0.525±0.213	1.691	1.113	2.569	0.014
**Acquaintance injured/died (n = 1656)^d^**					
No (n = 1463)		1.000			
Yes (n = 193)	0.447±0.219	1.564	1.018	2.403	0.041
**Timing to move into tents (n = 1820)^e^**	0.035±0.015	1.036	1.006	1.067	0.020
**Timing to resume classes (n = 1774)^f^**					
<1 week (n = 107)		1.000			
1–2 weeks (n = 53)	−0.757±0.503	0.469	0.175	1.258	0.133
3–4 weeks (n = 418)	−1.013±0.319	0.363	0.194	0.678	0.001
>4 weeks (n = 1196)	−1.202±0.298	0.301	0.168	0.539	0.000

Data represent the mean ± S.E. a) compared with male; b) compared with not feeling danger; c) compared with not witnessing earthquake related events; d) compared with all safe; e) compared with moved into tents within 2 days; f) compared with classes resumed in less than one week. OR: Odds ratio.

### Correlation between PTSD and depression

**Figure 2:**
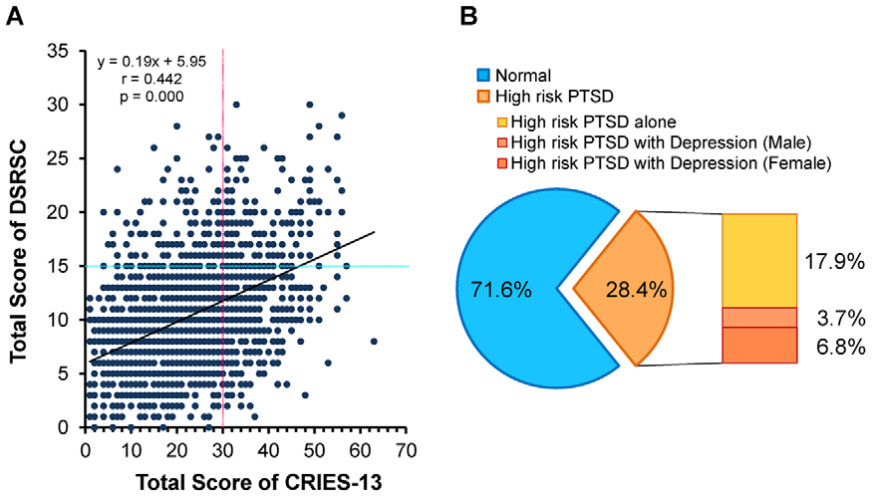
Correlation between CRIES-13 and DSRSC in junior middle school students 10 months after the Wenchuan earthquake. (A): There was a positive correlation (r = 0.442, p = 0.000) between the total scores from CRIES-13 and DSRCS. (B): The prevalence of a high-risk for PTSD was 28.4% (522 students) among all students. Of the 522 students with PTSD, the incidences of PTSD alone, male PTSD with depression, female PTSD with depression were 17.9%, 3.7%, and 6.8%, respectively.

There was a significant, positive correlation between CRIES-13 (PTSD) and DSRSC (depression) scores (**Figure 2A**), indicating an association between a high-risk for PTSD and a high risk for depression (**Figure 2A**). Furthermore, comorbid depression was found at a high rate in this high-risk for PTSD population (**Figure 2B**).

### The CHAID decision tree analysis of PTSD and depression

**Figure 3:**
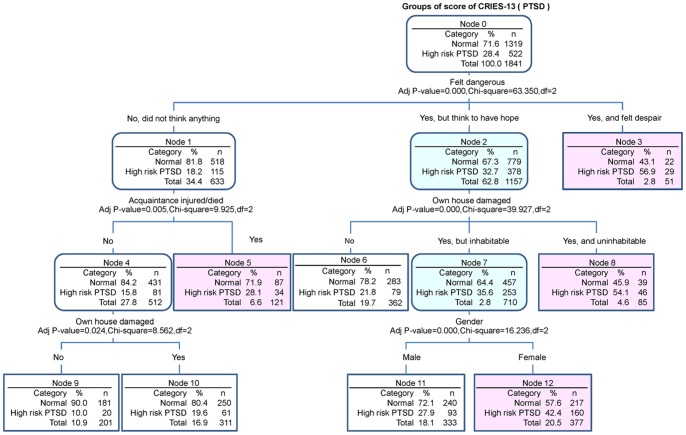
Chi-square automatic interaction detector decision trees for PTSD. The feelings of “danger” or “despair” were demonstrated to be the most sensitive risk factors. Compared to subjects who did not experience these feelings during earthquake, the prevalence of a high risk for PTSD was high in subjects who had felt despair or felt danger but who also had hope. Moreover, in subjects who had felt the danger, “damage to their own house” was the second major related risk factor. Of the subjects whose houses were destroyed, 54.1% had a high-risk for PTSD, a higher figure than seen for subjects whose houses were damaged (35.6%) or undamaged (21.8%).

The CHAID decision tree analysis of CRIES-13 (**Figure 3**) showed that a positive response to the question of whether students had “felt danger or felt despair” was the most sensitive indicator for estimating PTSD (p = 0.000). Compared with subjects who had not felt despair during the earthquake, the prevalence of a high risk for PTSD was high in subjects who had felt despair or felt danger but had hope (18.2% [115/633] vs. 56.9% [29/51] vs. 32.7% [378/1157]). Moreover, in subjects who had “felt danger”, a positive response to “Whether their own house was damaged” was observed as the second major risk factor for PTSD (p = 0.000). A high risk for PTSD was estimated from the 54.1% of the subjects whose house was destroyed, a higher figure than for subjects whose house was damaged (35.6%) or undamaged (21.8%). Our data also showed that, having an acquaintance die or be injured was an important indicator in subjects who had not felt despair during the earthquake (p = 0.005).

**Figure 4:**
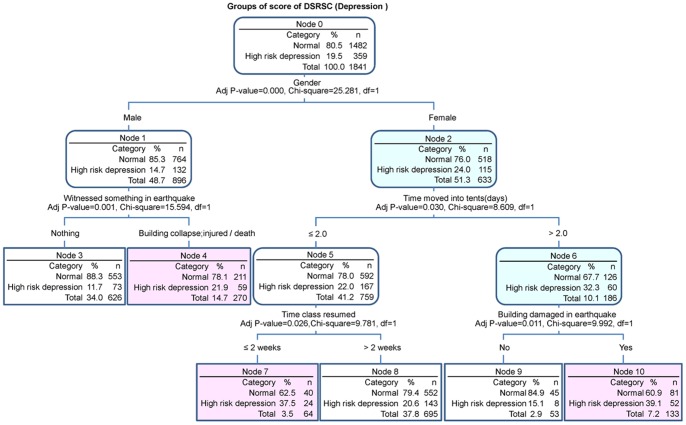
Chi-square automatic interaction detector decision trees for depression. Gender was the largest high-risk related factor for depression. Females showed a 24.0% risk for depression compared with males (14.7%). Additionally, “timing of the move into a tent” was the second major risk factor in females (p = 0.030), in contrast “witness experience during earthquake” was the second major risk factor in males (p = 0.001). The “timing of the resumption of classes” and “whether own house was damaged” were also important risk factors in females.

The CHAID decision trees analysis of DSRSC (**Figure 4**) showed that gender was the first high-risk factor for estimating depression (p = 0.000). Twenty four percent of females were estimated to be at high risk for depression compared with 14.7% of males. The “timing of the move to a tent” was the second major relevant factor in females (p = 0.030), while “witness experience during the earthquake” was the second major relevant factor in males (p = 0.001). Additionally, the time elapsed before the resumption of classes and whether their house was damaged, were also important relevant factors in females (p = 0.026 and p = 0.011, respectively).

## Discussion

The major finding of this study is the high prevalence of PTSD and depression in junior middle school students, in a rural disaster area, far from the earthquake epicenter. PTSD is the most common psychiatric disorder associated with natural disasters [5], [8], [18]. To date, most studies have been performed at the epicenter and its immediately surrounding areas. In this study, we examined the mental health status of a large sample (n = 2841) of junior middle school students in Ningqiang county, a rural town (Shaanxi Province, China), approximately 327 km from the earthquake epicenter. Although the scale of the disaster in Ningqiang county was lower than in Wenchuan county and its immediately surrounding areas, our results showed a high prevalence (28.4%) for PTSD development in our surveyed subjects. It is noteworthy that this prevalence is similar to that found in a previous study after the Wenchuan earthquake [18]. This study used the same PTSD measure scale to assess symptoms among junior middle school students in Mianzhu city (**Figure 1**) which is located near the earthquake epicenter. They found that the estimated high-risk for PTSD of 28.7% at one month, and 24.4% at 7 months after the Wenchuan earthquake. Several other researchers in China investigated the mental health status of children and adolescents in severe disaster areas, surrounding the epicenter, such as Dujiangyan city and Beichuan county (**Figure 1**) in the time period of 6 to 12 months after the Wenchuan earthquake. The incidence of PTSD was 15.8% in Dujiangyan, 6 months after the earthquake [14], 11.2% and 13.4% in Beichuan county, at 6 and 12 months respectively, after the earthquake [24] and 11.2%, 8.8%, 6.8% and 5.7% in Wenchuan county at 4, 6, 9, and 12 months respectively, after the earthquake [25]. Interestingly, Pynoos et al. [26] reported a positive correlation between the proximity to the epicenter of the quake and the severity of posttraumatic stress reactions in 231 children, after the 1988 Armenian earthquake. However, in our samples, the prevalence of a high-risk for PTSD was higher than that found in the areas immediately surrounding the epicenter. This difference may be attributable to some of the following experimental parameters: 1) The varied duration of time elapsed between the disaster and assessments; 2) The measure scales used; and 3) Whether a liberal or conservative threshold was used to define the ‘persistent’ symptoms of PTSD. Thus, it is difficult to make direct comparisons between these reports. It is likely that the following factors may contribute to the high prevalence of PTSD in our study: Firstly, the CRIES-13 scale used in our study is a screening scale of PTSD, and the results reflect estimated prevalence of a high-risk for PTSD. However, many studies on PTSD use criteria defined by DSM-IV, and examined adults rather than children [27]. Also, there is a tendency for the consequences of psychiatric trauma to be underestimated when thresholds are set [28], [29]. Recently, using CRIES-13 (cut-off score: 30), Ma et al. [5] reported on an estimated prevalence of high-risk PTSD at 11.3% in their sample, but only 2.6% of subjects met DSM-IV criteria for PTSD. Secondly, compared with the epicenter and surrounding areas, the effective social support, including the dispatch of mental health teams, arrived relatively late in the Ningqiang area. In the most severely affected areas, the government dispatched mental health teams quickly, as a matter of priority, relative to areas further away from the disaster. A report from Mianzhu city (**Figure 1**) which includes four counties severely affected by the earthquake showed that individuals who received better social support had significantly lower scores on the CRIES-13 scale [5]. In that study, the prevalence of probable PTSD was 11.3% (342/ 3,208 students) at 6 months after the earthquake [5]. Moreover, a long-term follow-up study after the Armenian earthquake showed that early intervention decreased the risk of chronic PTSD [30]. Thirdly, the discrepancy might be related to differences between urban and rural environments, such as low socioeconomic status.

A number of studies show that the degree of exposure to disaster is associated with the likelihood of PTSD [11], [31]–[34], and that female gender is a risk factor for the onset of PTSD after disasters [14], [35]–[37]. Our findings are consistent with these studies, indicating that the total score and three sub-scores (intrusion, avoidance, and high arousal factors) were significantly higher in females and the severe exposure group compared with males and the mild exposure group. Our results suggest that severe exposure was an important factor for the prevalence of PTSD, and that there is a gender difference in the ability to cope with stress after natural disasters. Gender differences were also reported in a study performed 9 months after the Kobe earthquake in Japan, in 1995 [37]. Furthermore, Goenjian et al. [38] performed a longitudinal study of PTSD on 511 adolescents using the PTSD Reaction Index (PTSD-RI), 3 and 32 months after the Parnitha earthquake, and observed higher PTSD-RI scores in girls. They found that, half of girls and a quarter of boys reported experiencing trauma reminders during the preceding month that made them think about the earthquake and 27% of girls versus 11% of boys reported experiencing two or more reminders during the previous month. On the other hand, Davidson et al. [34] reported that the prevalence rates of PTSD in natural disasters and major accidents were significantly higher in male students compared with female students (18.9% and 25.0% for males vs. 15.2% and 13.8% for females, respectively). These gender differences may in turn reflect the influence of gender on social roles in different cultures.

Major depressive disorder is also a common psychiatric disorder which frequently manifests after natural disasters, such as severe earthquakes, hurricanes, and wildfires [14], [20], [39]–[41]. In this study, we used DSRSC, a common assessment scale of depression, and found that the prevalence rate of depression in our sample was 19.5%. This prevalence was relatively lower than that estimated in the severe disaster areas, immediately surrounding the epicenter (24.5%) [14]. Our lower rate of depression may reflect differences in the magnitude of the earthquake, determining the extent of destruction, and loss of life and injuries. In our sample, students experienced less loss of loved ones and fewer injuries because the disaster effect in Ningqiang county was less severe. There may also be additional confounding factors in our study: 1) Ningqiang as a remote rural town, with strong community relationships. Living in tents after the earthquake might provide a relatively safe living environment where to some degree, the strong community spirit can be maintained, which would help to reduce stress; 2) our study was performed relatively late (10 months) after the earthquake. A previous study found that the prevalence rate of depression peaked at 6 months after the earthquake, and showed a tendency to reduce with the passage of time [42]. A number of studies found high prevalence rates of depression after earthquakes and tsunamis [8], [24], [43]. In contrast, our findings suggest that depression in children and adolescence is the lesser psychiatric disorder, in a rural area far from the earthquake epicenter.

In this study, we found high comorbidity of depression in the students with PTSD. Accumulating evidence shows that PTSD symptoms and depression are often comorbid [7], [8], [11], [24], [44], and the lifetime co-occurrence of other psychiatric disorders such as depression, with PTSD symptoms is high [7]. These data suggest a need for mental health support for children living in rural areas, even if they are far away from disaster epicenters.

We also examined the characteristics of high-risk factors related to PTSD and depression, using Logistic regression and CHAID decision trees. Similar to previous reports, factors such as “feeling despair” (OR: 5.0) or “feeling despair but also having hope” (OR: 2.2) during the earthquake, “having their own house destroyed” (OR: 2.9) or “having their house damaged but inhabitable” (OR: 2.0), “witnessing an injury or death” (OR: 2.2), “witnessing a building collapse” (OR: 1.9), and being female (OR: 1.7) were identified as high-risk PTSD factors. Using CHAID decision trees analysis, we found that “feeling despair during the earthquake” was the strongest related factor to PTSD, consistent with other natural disasters such as Tsunamis [43].

In our study, the main risk factors for depression were feeling despair during the earthquake (OR: 3.5), and being of female gender (OR: 1.8). It is also of interest that the early reopening of school classes was a critical risk factor for depression. Our study showed that reopening classes after a minimum time of one month after the earthquake reduced the risk of depression (OR: 0.3–0.47). Although, other researchers indicate that the severity of post-disaster depression could be predicted by the extent of loss of significant others and perceived threat and fear during the earthquake [14], [35], gender was the first critical risk factor selected from various high-risk factors for depression in our sample. This difference may be related to the low mortality in Ningqiang county. In addition, for females, delayed evacuation and early reopening of classes, while for males, witnessed events were important risk factors for depression. Early resumption of school after the earthquake is considered one of the main goals for Provincial Reconstruction. However, our results revealed that this is not necessarily the best policy. The early resumption of classes may promote additional psychological stress in students. To prevent depression, it may in fact be essential for children to spend as much time as possible with their families after an earthquake.

Unfortunately, there are some limitations to this study, the main one being that our survey was conducted 10 months after the earthquake, and no early data was collected. Thus, we could not evaluate acute stress reactions to trauma in this cohort. Furthermore, our sample included several students with ages higher than would typically be expected in junior middle school (16–20 years). This wider age range was due to two main phenomena: firstly, children from impoverished and rural areas often enroll in school at a later age and secondly, in China, the grade repetition system applied even in elementary and junior middle school.

### Conclusions

This study showed a high prevalence of PTSD and depression among junior middle school students in a rural area, 10 months after the Wenchuan earthquake. The prevalence of PTSD and comorbid depression was high among children and adolescents, even though their homes were not located in the severe disaster area, but 327 km from the epicenter. Our data indicates that mental health interventions are necessary for children and adolescents in rural areas, far from the epicenter to reduce the prevalence of PTSD and depression.
